# Crystal Structure of the Minimalist Max-E47 Protein Chimera

**DOI:** 10.1371/journal.pone.0032136

**Published:** 2012-02-28

**Authors:** Faraz Ahmadpour, Rodolfo Ghirlando, Antonia T. De Jong, Melanie Gloyd, Jumi A. Shin, Alba Guarné

**Affiliations:** 1 Department of Biochemistry and Biomedical Sciences, McMaster University, Hamilton, Ontario, Canada; 2 Laboratory of Molecular Biology, National Institute of Diabetes and Digestive and Kidney Diseases, National Institutes of Health, Bethesda, Maryland, United States of America; 3 Department of Chemistry, University of Toronto, Mississauga, Ontario, Canada; MRC National Institute for Medical Research, United Kingdom

## Abstract

Max-E47 is a protein chimera generated from the fusion of the DNA-binding basic region of Max and the dimerization region of E47, both members of the basic region/helix-loop-helix (bHLH) superfamily of transcription factors. Like native Max, Max-E47 binds with high affinity and specificity to the E-box site, 5′-CACGTG, both *in vivo* and *in vitro*. We have determined the crystal structure of Max-E47 at 1.7 Å resolution, and found that it associates to form a well-structured dimer even in the absence of its cognate DNA. Analytical ultracentrifugation confirms that Max-E47 is dimeric even at low micromolar concentrations, indicating that the Max-E47 dimer is stable in the absence of DNA. Circular dichroism analysis demonstrates that both non-specific DNA and the E-box site induce similar levels of helical secondary structure in Max-E47. These results suggest that Max-E47 may bind to the E-box following the two-step mechanism proposed for other bHLH proteins. In this mechanism, a rapid step where protein binds to DNA without sequence specificity is followed by a slow step where specific protein:DNA interactions are fine-tuned, leading to sequence-specific recognition. Collectively, these results show that the designed Max-E47 protein chimera behaves both structurally and functionally like its native counterparts.

## Introduction

The basic helix-loop-helix (bHLH) proteins are a widely distributed superfamily of transcription factors that regulate genes important for cell proliferation, differentiation and apoptosis [1]. These transcription factors comprise an N-terminal basic region (b) necessary for binding to a shared signature DNA-motif ( ^5′^CANNTG) and a C-terminal helix-loop-helix (HLH) region that mediates homo- or heterodimerization [2], [3]. Some members of the bHLH superfamily include additional structural motifs, such as the bHLHZ family that contains a C-terminal leucine zipper (Z) or the bHLHPAS family that includes a Per-Arnt-Sim (PAS) domain adjacent to the helix-loop-helix region.

The Myc oncoprotein is likely the best-studied member of the bHLHZ family. In response to cellular signals, Myc regulates many processes, including cell proliferation, growth and transformation, whereas deregulated expression of Myc increases apoptosis, genomic instability and angiogenesis [4]. Activation by Myc requires heterodimerization with Max, a bHLHZ transcription factor that serves to regulate other members of this superfamily [5], [6], [7]. In the absence of Max, Myc is incapable of binding to its target DNA sequence ( ^5′^CACGTG), known as the Enhancer-box (E-box). Conversely, Max readily homodimerizes and binds the E-box with high affinity [7]. Max also forms heterodimers with other bHLHZ proteins, including the Mad1 transcription factor. The Mad1/Max complex functions as a transcriptional repressor [8], [9], [10], [11] and, thus, it has been suggested that Myc/Max and Mad/Max complexes define a molecular switch regulating the cellular transition from a growth to a resting state.

Several crystal structures of bHLHZ proteins bound to their cognate E-boxes have been determined [12], [13], [14], [15], [16], [17]. While biochemical studies had originally proposed that binding of bHLHZ proteins to the E-box imposed significant bending of the DNA, the crystal structures revealed that bHLHZ proteins either do not bend or only mildly bend DNA. Remarkably, the conformations of all bHLHZ complexes solved to date are virtually identical, revealing the high structural conservation within this family of proteins. The basic region, responsible for DNA binding, defines the N-terminus of the first α-helix and in the absence of DNA. This helix is presumably unfolded, but becomes structured upon binding to its target DNA sequence [18], [19]. The helix-loop-helix subdomain forms a four-helix bundle that is responsible for dimer formation and specification of dimerization partners. In the structure of wild-type Max bound to DNA, the leucine zipper following the helix-loop-helix subdomain is not well defined, suggesting that this motif improves partner specificity rather than strengthening dimerization *per se* [12]. Comparison of the Myc/Max and Max/Max structures explains why Max, but not Myc, can form both homo- and heterodimers [17].

Since the biological functions of Myc depend on the Myc/Max/Mad network [20], regulating the interaction of Myc with its binding partner Max has become an attractive therapeutic target. Omomyc, a variant of Myc encompassing four point mutations within its leucine zipper, interacts with Myc. These Myc/Omomyc heterodimers do not bind DNA, but sequester Myc into inactive complexes [21]. The positive effects associated with introducing Omomyc in a Myc-induced tumorigenesis mouse model are dependent on the presence of oncogenic Myc, supporting the idea that sequestration of Myc is of therapeutic value [22]. In recent years, an alternative dominant negative strategy using minimalist proteins designed to block binding of the Myc/Max complex to the E-box has been devised. One of these proteins, Max-E47, encompasses the basic region of Max and the helix-loop-helix of E47, which is also a member of the bHLH superfamily but does not possess a leucine zipper [23]. Like Max, E47 forms homo- and heterodimers with other bHLHZ proteins [3]. The Max-E47 chimera binds to E-box DNA with affinity similar to the Max homodimer and outcompetes Max binding to the E-box in the yeast one-hybrid assay [23].

We have solved the crystal structure of the Max-E47 homodimer and characterized its oligomeric state. The basic region of Max-E47 forms an α-helix that closely resembles the conformation seen in other Max bHLHZ structures bound to DNA, suggesting that Max-E47 forms a structured dimer in the absence of DNA. Accordingly, Max-E47 monomers were not detected by analytical ultracentrifugation, even at low micromolar concentrations, demonstrating that Max-E47 exists predominantly as a dimer in solution. Circular dichroism analysis revealed that Max-E47 becomes more ordered upon DNA binding, suggesting that the basic region of the protein becomes stabilized in a helical conformation upon DNA binding or at increasing protein concentrations. Similar to the bHLHZ transcription factor USF [24], Max-E47 becomes more ordered whether in the presence of specific E-box DNA or nonspecific DNA, indicating that it may also follow a two-step binding mechanism, wherein a rapid and non-specific association with DNA is followed by a slow conformational change induced by E-box recognition.

## Materials and Methods

### Protein Expression and Purification

The Max-E47 hybrid was designed as described earlier [23]. In brief, Max-E47 was generated by fusing the DNA binding basic region of Max (residues 22 to 36) to the helix-loop-helix dimerization region of E47 (residues 349 to 400) and subcloned into the pET28a(+) vector using the NcoI and XhoI restriction sites. For protein overproduction, BL21(DE3) cells were transformed with the plasmid encoding Max-E47 (pAG8349) and grown in the presence of kanamycin (100 µg/mL) to an OD_600_ of approximately 0.7 at 37°C with orbital agitation. The cultures were then chilled on ice with agitation, and protein over-production was induced by addition of 0.1 mM IPTG. The cells were grown for 5 hours at 25°C with agitation and subsequently harvested by centrifugation at 5,000 *g* for 10 minutes. Cell pellets were washed with ice-cold phosphate buffered saline and stored at −80°C. The two cloning variants of Max-E47, Max-E47Y and Max-E47YF (pAG8474 and pAG8475, respectively) [23], were produced in a similar manner.

Max-E47, Max-E47Y and Max-E47YF were found in the insoluble fraction of the cell lysates and, hence, all purification steps were conducted under denaturing conditions at room temperature. Cells pellets were resuspended in 20 mL per liter of cells in buffer A (20 mM Tris pH 8, 0.3 M NaCl, 10 mM 2-mercaptoethanol, 10% (v/v) glycerol, 8 M urea and 0.03 M imidazole) supplemented with 25 µL lauryldimethylamine oxide (LDAO) and a cocktail of protease inhibitors containing 1 mM PMSF, 0.7 µg/mL Pepstatin A, 5 µg/mL leupeptin and 1 mM benzamidine. Cells were lysed by sonication and the lysate cleared by centrifugation at 40,000 *g* for 40 minutes. The supernatant was collected, filtered and loaded onto a HiTrap Ni-affinity column (GE Healthcare) pre-equilibrated with buffer A. The column was washed with buffer A supplemented with 0.06 M imidazole to remove contaminants bound to the column and Max-E47 was subsequently eluted with buffer A containing 0.3 M imidazole.

### Protein refolding

Max-E47 was refolded by a combination of dilution and dialysis to remove urea. Fractions containing Max-E47 were pooled after collection from the HiTrap Ni-affinity column and diluted into buffer B (20 mM Tris pH 8, 0.3 M NaCl, 10 mM 2-mercaptoethanol and 10% (v/v) glycerol) to a final concentration of 1 M urea and dialyzed against buffer B containing 1 M urea for 3 hours at room temperature. This sample was subsequently dialyzed against buffer B containing 0.5 M urea and against buffer B containing 0.25 M urea at 4°C overnight. The refolded sample was then allowed to reach room temperature and loaded on an SP-Sepharose column (GE Healthcare) pre-equilibrated with buffer C (20 mM Tris pH 8, 0.1 M NaCl, 10 mM 2-mercaptoethanol and 10% (v/v) glycerol). Max-E47 was eluted from the column using a step gradient from 0.1–1 M NaCl (Max-E47 elutes at 0.6 M NaCl). Protein containing fractions were pooled and concentrated to 3 mg/mL in storage buffer (10 mM Tris pH 8, 0.1 M NaCl, 5 mM 2-mercaptoethanol and 5% (v/v) glycerol). The oligomeric state of the protein was assessed by size-exclusion chromatography using a Superdex-75 column (GE Healthcare) and the lack of aggregates was confirmed by dynamic light scattering on a Nano-S Zetasizer (Spectra Research Corporation) using a 12 µL cuvette. In contrast to Max-E47, the Max-E47Y and Max-E47YF variants had to be stored at room temperature to prevent protein aggregation.

### Crystallization, Data Collection, Structure Determination and Refinement

Initial crystals were obtained with the sparse matrix anions suite (Qiagen) using the vapor diffusion method on sitting drops. Optimal crystals grew in 0.1 M sodium acetate anhydrous pH 4.6, 3.2–3.5 M sodium nitrate and 5% glycerol at 4°C and reached their maximum size in one to three weeks. Prior to flash-freezing them in liquid nitrogen, crystals were cryo-protected by addition of 25% (v/v) glycerol to the crystallization solution.

A complete data set was collected at the ×25 beam line in NSLS, Brookhaven National Laboratory (Upton, NY). Data were indexed, processed and merged using HKL2000 [25]. The initial phases were determined by molecular replacement using PHASER [26] and the E47 monomer as searching model (PDB ID: 2QL2, [16]). The final structure was obtained by alternating cycles of manual building in COOT with refinement in phenix.refine using TLS [27], [28]. The final model has 96.4% of residues in most favored regions, and none in the disallowed regions of the Ramachandran plot as judged by MolProbity [29]. Accessible surface areas were calculated using the areaimol program in CCP4 with a solvent probe radius of 1.4 Å [30]. Atomic coordinates and structure factors of Max-E47 have been deposited in the Protein Data Bank under accession code 3U5V.

### Analytical Ultracentrifugation

To remove protein aggregates due to freezing and thawing of the sample, as well as to exchange the buffer prior to analytical ultracentrifugation, samples of Max-E47 or Max-E47YF were purified by size-exclusion chromatography using a Superdex75 column (GE Healthcare) pre-equilibrated with ultracentrifugation buffer (20 mM Tris pH 8.0, 0.15 M NaCl and 10 mM 2-mercaptoethanol). Sedimentation velocity experiments, as well as sedimentation equilibrium experiments, on Max-E47 were carried out in ultracentrifugation buffer. To remove remaining traces of glycerol, the protein eluted from the Superdex75 column was loaded a second time onto the Superdex75 column.

Sedimentation velocity experiments were conducted at 20.0°C on a Beckman Coulter ProteomeLab XL-I analytical ultracentrifuge. 400 µL of each of the samples at various concentrations were loaded into 2-channel centerpiece cells, allowed to equilibrate at 20.0°C under vacuum for at least 4 hours and subsequently analyzed at 50,000 rpm over a period of 12 hours. Scans were collected using the Rayleigh interference optical detection system at seven-minute intervals. Data were analyzed in SEDFIT 12.1b [31] in terms of a continuous c(*s*) distribution of Lamm equation solutions using an uncorrected *s* range of 0.0–5.0 S with a resolution of 100 and a confidence level of 0.68. In all cases, excellent fits were obtained with interference root mean square deviations values ranging from 0.002–0.005 fringes. Solution densities ρ, viscosities η and protein partial specific volumes *v* were calculated in SEDNTERP 1.09 [32]. In the case of buffers containing 5% (v/v) glycerol, solution densities were measured experimentally at 20.0°C on a Mettler-Toledo DE51 density meter; the exact glycerol concentration was thus determined and the corresponding viscosity calculated in SEDNTERP 1.09. Sedimentation coefficients *s* were corrected to *s_20,w_*. Interference signal increments ε_J_, used for the estimation of sample concentrations, were calculated using ε_J_ = (M/1000) (*dn*/*dc*)/λ, where M is the molecular mass, λ the wavelength in cm (655×10^−7^ cm) and *dn*/*dc* the refractive index increment. A value of 0.185 cm^3^/g was assumed for *dn*/*dc*.

Sedimentation equilibrium experiments were conducted at 16.0°C on a Beckman Coulter ProteomeLab XL-I analytical ultracentrifuge in ultracentrifugation buffer. 160 µL of each of the samples at various concentrations were loaded into mechanically aged 2-channel centerpiece cells [33] and analyzed at various rotor speeds ranging from 14,000 to 35,000 rpm. Scans were collected using the Rayleigh interference optical detection system at six-hour intervals until sedimentation equilibrium was reached, as determined by WinMATCH v0.99. Equilibrium was reached within 54 hours, at which point the rotor speed was increased to the next higher speed. Water blanks previously obtained for the mechanically aged cells were subtracted from the experimental data prior to analysis in SEDPHAT8.2 [34]. Various models, including a species analysis and a monomer-dimer self-association, with and without mass conservation, were used to analyze the data. Time-independent (TI) noise corrections were implemented when these did not correlate with the best-fit model [35]. Excellent fits were obtained for data presented with interference root mean square deviations values ranging from 0.003–0.005 fringes. Experiments were carried out using long column lengths and various rotor speeds. The long column lengths and meniscus depletion were used in order to identify Max-E47 monomer, if present, and discriminate between the smallest species and higher order aggregates. Furthermore, the lowest rotor speeds were designed to discriminate between the Max-E47 tetramer and smaller mass complexes.

### Circular Dichroism Spectroscopy

250 µL samples were prepared with 10 µM protein monomer in 15.1 mM Na_2_HPO_4_, 4.9 mM KH_2_PO_4_, 50 mM NaCl, pH 7.4, and 10 µM duplex DNA where appropriate (i.e. 1∶2 ratio of protein dimer:DNA duplex). For native MaxbHLHZ, the temperature-leap tactic was used to ensured folded, functional proteins for circular dichroism (CD) measurements [36]. Samples, including the buffer control without protein, were prepared and incubated overnight at 4°C, followed by approximately 20 minutes incubation at room temperature. For Max-E47, the protein stock was stored in 20 mM Tris pH 8, 0.1 M NaCl, and an equivalent amount of this storage buffer was added to the buffer blank. Samples were incubated overnight at room temperature, as aggregation had been observed at 4°C. CD data was collected on an Aviv 215 spectrometer with a Suprasil, 1 mm path-length cell (Hellma, Plainview, NY) at 22°C. Spectra were acquired between 190 and 300 nm at 0.2 nm increments and a sampling time of 0.2 seconds. Each spectrum was the average of two scans with the average buffer (and DNA where appropriate) control spectrum subtracted. Data obtained were not smoothed. Protein helix content was calculated using the method described by Chau and coworkers [37].

## Results and Discussion

### Structure of Max-E47

Crystals of Max-E47 grew at 4°C and reached their maximum size in about three weeks. The crystals belong to the I2_1_2_1_2_1_ space group, have low solvent content and diffract X-rays to 1.7 Å resolution (Table 1). The structure was determined by molecular replacement using the E47 monomer from the E47/NeuroD1 structure (PDB accession code: 2QL2, [16]) as searching model and residues 7 to 68 could be readily traced in the electron density maps.

**Table 1:** Data collection and refinement statistics.

**Data Collection**	
Space group	I2_1_2_1_2_1_
Cell Dimensions (Å, °)	37.48, 49.47, 74.1, 90, 90, 90
Wavelength (Å)	1.1
Resolution (Å)^a^	40–1.7 (1.73–1.7)
Completeness (%)^a^	99.7 (98.5)
Redundancy^a^	8.9 (4.6)
Rmerge (%)^a^	6.3 (64.7)
I/σ(I)^a^	32.1 (2.1)
Wilson B-value (Å^2^)	24.2
Solvent content	47%
**Refinement**	
Resolution (Å)	23.3–1.7
Number of reflections (work/test)	7,197/353
Rwork/Rfree (%)	20.6/23.8
Number of atoms (protein/solvent)	514/34
R.m.s.d. bond length (Å)	0.003
R.m.s.d. bond angles (°)	0.567
Ramachandran Plot (%) (Favored/additional/disallowed)	96.4/3.6/0
Maximum likelihood coordinate error	0.14

a Data in the highest resolution shell are shown in parentheses.

**Figure 1:**
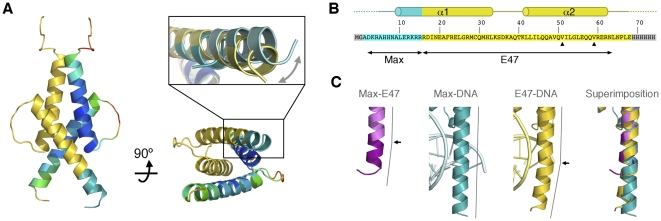
Structure of Max-E47. (**A**) Orthogonal views of the Max-E47 structure shown as a ribbon representation with one protomer colored as a rainbow by B-factor (10 (blue)≤B≤70 (red)) and the other protomer shown with basic region from Max in blue and the helix-loop-helix region from E47 in yellow. The inset highlights the bending of helix a1 in Max-E47 (blue-yellow) towards the symmetry axes of the dimer. The structures of MaxbHLHZ (light blue) and E47bHLH (light yellow) are shown as a reference. (**B**) Sequence of the Max-E47 chimera with the basic regions from Max and E47 colored as in panel (**A**). Sequences added during cloning are shaded in grey. The secondary structure elements are indicated above the sequence, with the disordered regions shown as dashed lines. The native residues of Max and E47 are indicated underneath and the two mutations that create the Max-E47Y and Max-E47YF cloning variants are marked with arrows. (**C**) Detail of the conformational changes in helix a1 induced by DNA binding. From left to right: Max-E47 (Max and E47 portions colored in purple and pink, respectively), MaxbHLHZ bound to DNA (teal), E47bHLH bound to DNA (yellow) and a superimposition of the three structures. The helical axes are indicated as grey lines beside each structure and kinks in the helices are marked with black arrows.

The asymmetric unit of the crystal contains one monomer of Max-E47, though a Max-E47 dimer forms by crystallographic symmetry (Figure 1A, 1B). Despite the absence of its cognate DNA target, the overall structure of Max-E47 is virtually identical to those of other bHLH and bHLHZ dimers [13], [15], [16], [38]. This finding was somewhat unexpected because it had been previously shown that addition of polyions, such as DNA, assists dimerization and increases the structural organization of bHLH and bHLHZ proteins [39]. However, the conformation of helix α1 is different from that of other bHLHZ structures determined in the presence of DNA (Figure 1).

In contrast to the structure of Max bound to DNA where the basic region of helix α1 is straight, the corresponding region of Max-E47 kinks at residue Arg16, thereby defining a concave inner groove that effectively narrows the access to the DNA-binding groove (Figure 1C). A kink in helix α1 is also present in the structure of E47 bound to DNA; however, it occurs a turn earlier (at residue Ala343) and should not interfere with DNA binding (Figure 1C). The bending of the basic region of Max-E47 suggests that DNA binding would force this region of the protein to flare away from the symmetry axes in order to recognize the E-box. In a simple analogy, the basic region of the Max-E47 may work like a binder clip that must be opened in order to hold an E-box. While we cannot rule out that crystal packing induces this conformational change, the basic region of Max-E47 does not engage in any packing interactions and, hence, this possibility seems unlikely.

**Figure 2:**
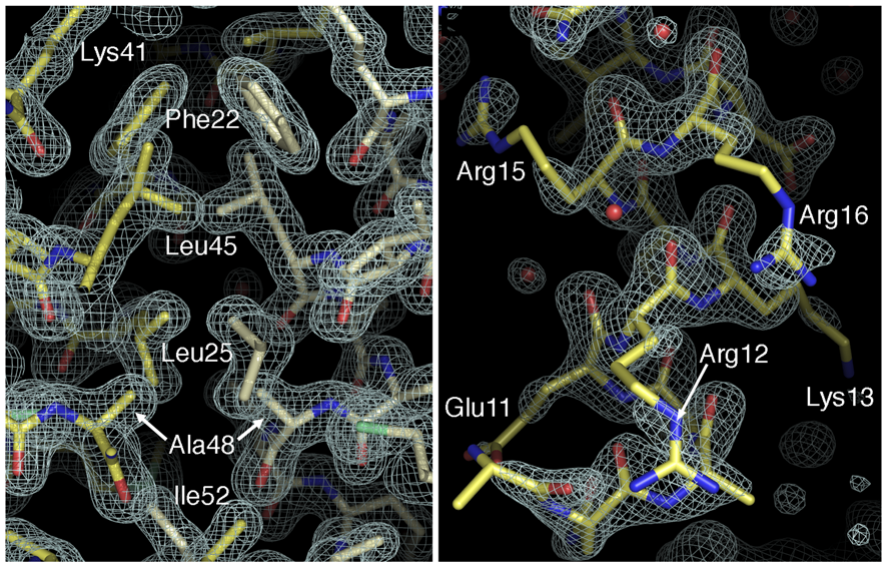
Electron density maps of Max-E47. Detailed view of the dimerization interface (**A**) and the basic region of Max-E47 (**B**). Composite omit electron density maps contoured at 1.5σ are shown as a white mesh. The two protomers of the Max-E47 dimer are shown as yellow and white color-coded sticks.

The helix-loop-helix region of Max-E47 is perfectly defined in the electron density maps and defines the characteristic parallel, left-handed four-helix bundle from this superfamily of transcription factors (Figure 2A). Conversely, only the main chain of the basic region is well defined in the electron density maps, while side chains show weak and fragmented electron density indicating their flexibility in the absence of its DNA target (Figure 2B). Similar to other structures of bHLH dimers, the loop connecting helices α1 and α2 is also more flexible than the rest of the protein, as indicated by the quality of the electron density maps and the higher B-factor values of this region (Figures 1A and 2B).

### Max-E47 is a dimer in solution

**Figure 3:**
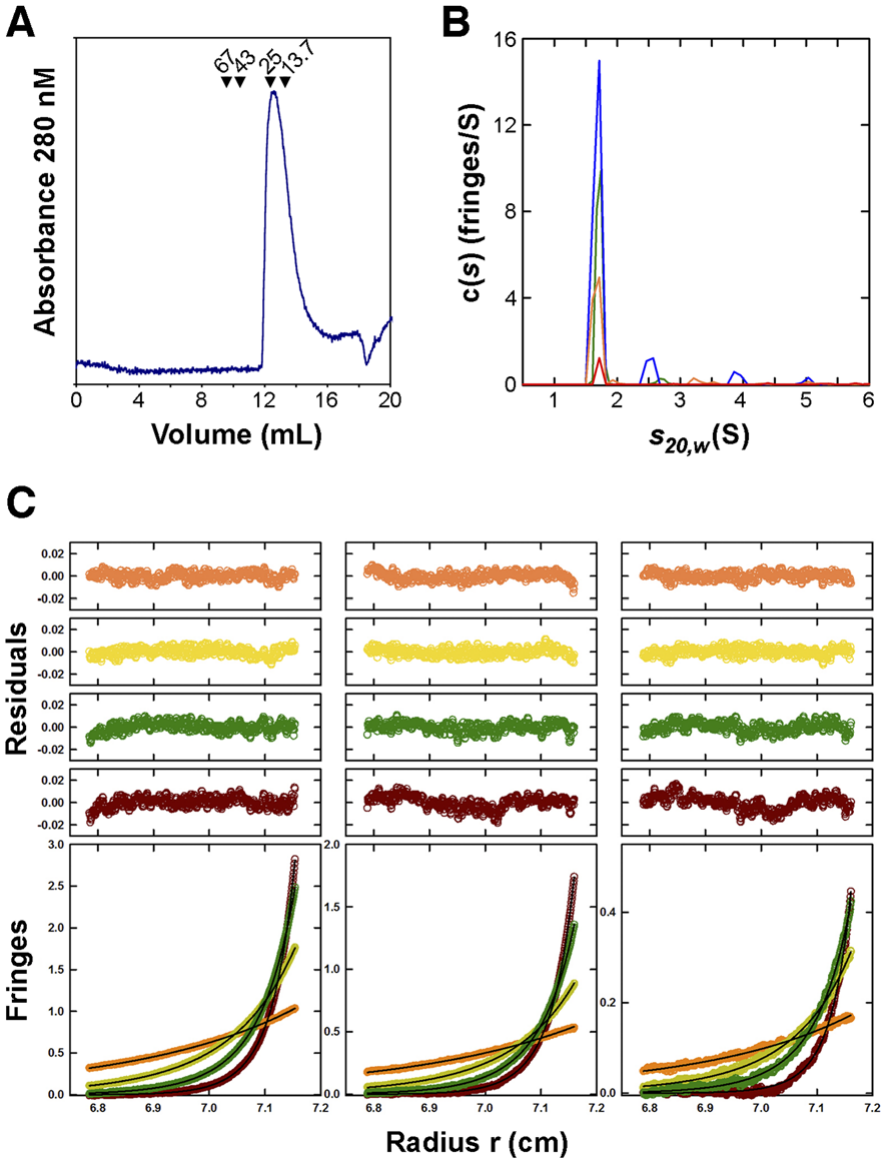
Max-E47 is a dimer in solution. (**A**) Size exclusion chromatography profile of Max-E47 over a Superdex75 column (GE Healthcare). Max-E47 elutes at a volume consistent with a dimer as reflected by the elution volumes of the molecular weight markers (albumin, 67 kDa; ovoalbumin, 43 kDa; chymotrypsinogen A, 25 kDa; and ribonuclease A, 13.7 kDa). (**B**) Continuous *c(s)* distributions obtained from sedimentation velocity data collected at 50 krpm, for Max-E47 (left) in 20 mM Tris pH 8.0, 0.1 M NaCl, 10 mM 2-mercaptoethanol and 5% (v/v) glycerol at loading concentrations of 3 (red), 22 (orange), 60 (green) and 130 (blue) mM. A major species is observed at 1.70 S representing a Max-E47 dimer, based on a best-fit molecular mass of 17.7±0.3 kDa (M_calc_ monomer = 9.066 kDa) obtained for this species in the absence of glycerol. (**C**) Sedimentation equilibrium profiles for Max-E47 at 16.0°C plotted as a distribution of the interference fringe displacement *vs.* radius at equilibrium. Data were collected at 14 (orange), 21 (yellow), 28 (green) and 35 (brown) krpm and loading concentrations of 25 (left panel), 10 (center panel) and 5 mM (right panel). The solid lines show the best-fit analyses in terms of two non-interacting species, returning molecular masses of 17.6 and 31.8 kDa and indicating the presence of both Max-E47 dimers and tetramers. The corresponding residuals for these best-fit analyses are shown in the plots above. Statistically indistinguishable fits (within 90% confidence intervals) were obtained when data were modeled in terms of a mixture of non-interacting Max-E47 dimers and tetramers. In these cases corrections for the time-invariant noise were not carried out.

Purified Max-E47 elutes from a size-exclusion column at a volume consistent with the presence of a dimer (Figure 3A). Accordingly, sedimentation velocity experiments revealed that Max-E47 is predominantly dimeric in solution over a broad concentration range (3–130 µM), although tetramers are also detected (Figure 3B). Interestingly, tetramers have not been previously detected for E47 [40], [41]. Sedimentation equilibrium data, modeled in terms of two non-interacting species (the simplest model that fit the data), returned molecular masses of 17.6 and 31.8 kDa indicating the presence of both Max-E47 dimers and tetramers (Figure 3C). These species do not appear to be in chemical equilibrium and simple mass conservation constraints indicate that the tetramer represents ∼17% of the total loading signal, thus confirming that Max-E47 is predominantly dimeric in solution. There was no evidence for any Max-E47 monomer, even at the highest rotor speeds.

**Figure 4:**
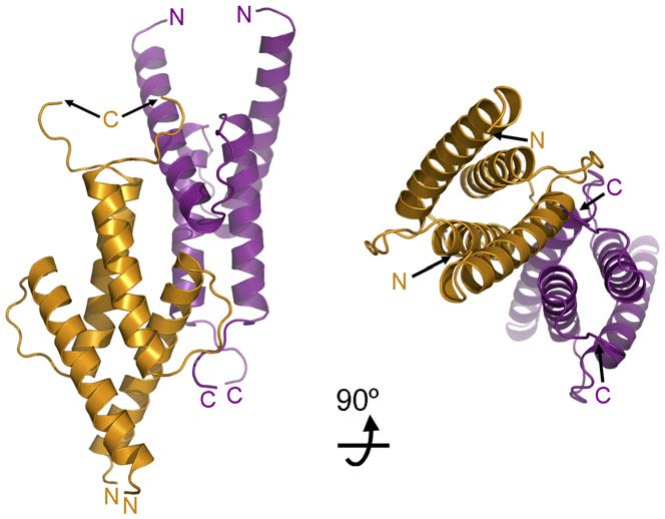
Oligomerization of the Max-E47 dimer. The crystal packing of Max-E47 suggests that dimers of Max-E47 (shown as orange and purple ribbon diagrams) can associate through crystallographic symmetry to form tetramers.

The formation of functional bHLHZ tetramers was first documented for the DNA binding domain of upstream stimulatory factor (USF) [14] and later visualized in the structures of the Max homodimer and the Myc/Max heterodimer [12], [17]. Indeed, it has been proposed that the functional unit of USF is the homotetramer [14], [24]. In the Myc/Max structure, the head-to-tail association of the individual leucine zippers of each heterodimer results in the formation of an antiparallel four-helix bundle heterotetramer. However, formation of the Myc/Max heterotetramer depends on the presence of the leucine zipper and can only be supported by the heterodimer, as it is stabilized through electrostatic interactions between the Max and Myc subunits in adjacent heterodimers. Therefore, the Max-E47 homotetramer detected by analytical ultracentrifugation is probably unrelated to the heterotetramer formed by the Myc/Max complex. The packing in the Max-E47 crystals reveals the presence of an intimate dimer-of-dimers arrangement that occludes 1,760 Å^2^ from the solvent – a significant area considering that dimerization occludes 3,120 Å^2^. Similar to the Myc/Max heterotetramer, the two dimers associate in a head-to-tail fashion, and the surface is stabilized by the interaction amongst the α2 helices of the two dimers (Figure 4).

**Figure 5:**
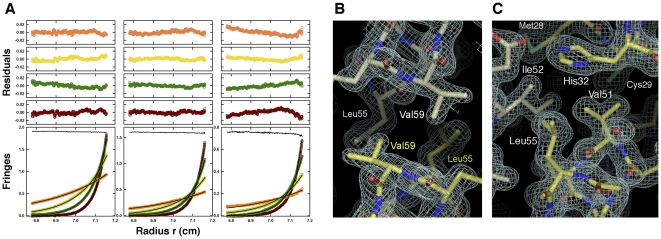
Max-E47YF is a monodisperse dimer in solution. (**A**) Sedimentation equilibrium profiles for Max-E47YF at 16.0°C plotted as a distribution of the interference fringe displacement *vs.* radius at equilibrium. Data were collected at 14 (orange), 21 (yellow), 28 (green) and 35 (brown) krpm and loading concentrations of 20 (left panel), 10 (center panel) and 5 µM (right panel). The solid lines show the best-fit analyses in terms of a single ideal solute with mass conservation constraints, returning a molecular mass of 18.8±0.3 kDa, and demonstrating that Max-E47YF is a monodisperse dimer (M_calc_ monomer = 9.179 kDa). The corresponding residuals for this best-fit are shown in the plots above. The best-fit time-invariant noise is also shown in each plot shifted by +1.9 (left), +1.6 (center) and +0.75 (right) fringes. Attempts to fit these data in terms of a MaxE47-YF monomer-dimer self-association indicate dimerization affinities tighter than 1 nM with a 95% confidence upper limit of 30 nM. (**B**) Detailed view of the 2Fo-Fc electron density map (contoured at 1 σ) around Val59. (**C**) Detailed view of the 2Fo-Fc electron density map (contoured at 1 σ) around Val51.

The Max-E47YF cloning variant, which possesses two point mutations in the helix-loop-helix region of the protein (Max-E47-V51Y/V59F [23]), also behaves primarily as a dimer in solution (Figure 5A). In this case, sedimentation equilibrium data were best fit in terms of a single species having a molecular mass of 18.8±0.3 kDa. Attempts to model these data in terms of a reversible monomer-dimer equilibrium self-association returned a *K*_d_ smaller than 1 nM and a 95% confidence upper limit of 30 nM. These data are consistent with the sole presence of dimers, and unlike Max-E47, no evidence for tetramers was observed. In this variant, Val51 and Val59 are mutated to Tyr and Phe, respectively, a change that should presumably weaken the stability of the dimer. The side chain of Val59 resides at the dimer interface and, hence, substitution by a larger side chain forces the dimer interface to breathe (Figure 5B). The effect of replacing Val51 by a larger aromatic residue is less clear. Val51 sits atop the four-helix bundle within a hydrophobic pocket defined by Cys29, His32 and Leu33 from helix α1, and Ile52 and Leu55 from helix α2 (Figure 5C). Mutation of Val51 to Tyr should widen this pocket and this, in turn, should destabilize the dimer. However, since Max-E47-YF monomers were not detected by sedimentation equilibrium under meniscus depletion conditions (Figure 5A), we presume that the dimerization dissociation constant of Max-E47 is likely in the low nanomolar range. Collectively, this data suggests that Max-E47 could bind to its cognate DNA site as a dimer, although we cannot rule out the possibility that Max-E47 exhibits monomer-dimer equilibrium at concentrations below the detection limits of our experiment.

### The helical content of Max-E47 increases upon DNA binding

It has been previously proposed that bZIP and bHLHZ transcription factors exist in monomer-dimer equilibrium in solution, and that the basic region is predominantly unstructured in the absence of DNA. Upon binding of two bHLHZ monomers to their cognate DNA target, folding of the basic regions is triggered and dimerization is enhanced [39], [41], [42]. Supporting the idea that DNA enhances the folding of the basic region, the majority of bHLHZ crystal structures have been determined in the presence of DNA [12], [13], [14], [16], [17]. The structure of the ATF4- C/EBPβ heterodimer, from the bZIP family of proteins, was determined in the absence of DNA [43]. In this structure, the basic region of ATF4 adopts a helical conformation while that of C/EBPβ is mostly disordered, reinforcing the idea that DNA enhances, but does not govern helix formation. Despite the absence of DNA, most of the basic region of Max-E47 adopts a helical structure. Residues Ala5-Arg15 in the basic region of Max-E47 are ordered in the crystal structure, though the side chains deemed important for E-box recognition are poorly defined in the electron density maps presumably due to their increased flexibility (Figure 2). The fact that only the first turn of this helix (residues Ala1–Arg4) is disordered suggests that binding to the E-box promotes stability of the Max-E47 structure, but DNA binding is not a requirement for induction of helical conformation in the basic region.

To probe this idea, we assessed the secondary structural content of Max-E47 in the presence or absence of DNA by circular dichroism (Table 2 and Figure S1). In the absence of DNA, Max-E47 helicity was 41% but underwent a modest folding transition upon addition of DNA. Interestingly, the sequence of the DNA had only a minor effect on the structure of Max-E47, as both E-box and non-specific DNA caused similar increases in helicity (to 56% and 51%, respectively). This folding transition corresponds to 7–10 residues becoming ordered upon addition of DNA, a change that can be correlated with the ordering of the N-terminal portion of the basic region (Ala1–Arg4) in the two protomers of the dimer. A similar trend was observed for native MaxbHLHZ, which was 49% helical in the absence of DNA, increasing to 66% in the presence of non-specific DNA or 67% when its cognate E-box was added. Therefore, addition of duplex DNA moderately increased protein secondary structure of Max-E47 and MaxbHLHZ, regardless of DNA sequence. Their specific DNA binding also parallels this comparable behavior, as both MaxbHLHZ and Max-E47 display identical high-affinity *K*_d_ values with the E-box (Table 2).

**Table 2:** Circular dichroism and DNA binding affinities.

Protein	% Helicity	% Helicity (with NS DNA^a^)	% Helicity (with E-Box^b^)	K_d_ (nM) with E-Box^b^
Max	49	66	67	14.3±7.9^c^
Max-E47	41	51	56	15.3±1.6^c^
E47^d^	50	—	80	—

a NS DNA is non-specific DNA (5′-TGCAGGAATTCCAAGGTGAAGGTT);

b E-box DNA used in CD is 5′-TGCAGGAACCACGTGGTGAAGGTT. For previously published K_d_ values obtained by fluorescence anisotropy, the same DNA sequence was used, except that the forward oligonucleotide was labeled at the 5′ end with 6-carboxyfluorescein (6-FAM);

c from [23];

d from [41]. Note that our CD data is presented for 10 µM monomeric protein concentrations, whereas Wendt et al. used 20 µM E47 monomer [41].

Folding transitions upon non-specific DNA binding have also been observed for other native bHLH and bHLHZ proteins such as MASH-1, Pho4, and the bHLHZ domain of USF [14], [18], [44], [45]. Based on steady state fluorescence analysis, a two-step binding mechanism for DNA binding of USF where fast association of the protein:DNA complex is followed by a slow conformational rearrangement that could involve adjustment of protein side chains to favor specific interactions with DNA was proposed [24]. This DNA-binding mechanism could explain the minimal differences in secondary structure observed for Max-E47 whether in the presence of non-specific or specific DNA (Table 2). All bHLHZ proteins analyzed to date exhibit high-affinity binding to sequence-specific DNA and, in some cases, even the dissociation constants for nonspecific DNA are lower than the relatively high protein concentrations required for CD spectroscopy (*K*_d_ values of 0.1–50 nM for protein:non-specific DNA complexes compared to 1–100 µM protein concentrations required for CD) [18], [44], [45]. Max-E47 and MaxbHLHZ showed no detectable binding to non-specific DNA up to 2 µM monomeric concentration by fluorescence anisotropy [23]. Unlike the fluorescence anisotropy studies, the CD measurements were performed with 10 µM protein, lower ionic strengths and the absence of competitors; it is, therefore, possible that Max-E47 binds to nonspecific DNA under these conditions.

### Potential binding mechanisms of Max-E47 to DNA

Two distinct models have been proposed for recognition of the E-box by bHLHZ proteins. Kohler *et al.* proposed that a monomer pathway, described as sequential binding of monomers to DNA followed by dimerization, would lead to enhanced specificity by avoiding kinetic trapping of pre-formed dimers bound at non-specific sites [42]. Meanwhile, Sha *et al.* proposed a two-step binding mechanism characterized by the fast and unspecific association of USF to DNA, followed by a slow conformational rearrangement that could involve adjustment of protein side chains to favor specific interactions with DNA [24]. We have not been able to detect monomers of Max-E47 and, while we cannot exclude that monomers exist at lower concentrations, our results support that Max-E47 achieves DNA-binding specificity through the latter two-step pathway. Supporting this mechanism, NMR studies of Pho4 showed very little difference in secondary structure and backbone dynamics of the basic region whether specific or non-specific DNA was present [18]. These data suggest that Pho4 may bind DNA through favorable electrostatic interactions between the negative DNA backbone and positive basic region, thereby triggering helix formation in the basic region. Subsequent, stable DNA binding is only achieved when specific side chains within the basic region recognize their target DNA sequence. A similar mechanism has been proposed for Max binding to DNA [19], [46]. Both Sauve *et al.* and Cohen *et al.* presented data consistent with a pathway where rapid, weak protein:DNA binding and formation of secondary structure are followed by slower fine-tuning conformational change of the protein side chains, upon location of the specific DNA target [18], [24]. Thus, the helical folding transition of Max-E47 measured by CD may reflect the rapid association of Max-E47 with DNA rather than correlate with specificity of DNA binding.

### Conclusions

This work reveals that the Max-E47 chimera retains the structural organization of the bHLH superfamily and has oligomerization properties similar to E47. Our analytical ultracentrifugation studies show that Max-E47 behaves as a dimer even at low micromolar loading concentrations and the modest conformational changes measured by circular dichroism suggest that Max-E47 may target its intended E-box site by a pathway similar to that exhibited by bHLHZ proteins such as Max, Pho4 and USF. Collectively, these results reveal that this non-native, engineered protein chimera designed by fusing the basic region of Max and the HLH region of E47 behaves both structurally and functionally like its native counterparts, thereby providing a molecular tool to modulate the Myc/Max/Mad network.

## Supporting Information

Figure S1 **Circular dichroism.** Spectra of (**A**) Max-E47 and (**B**) MaxbHLHZ in the absence of DNA (green), with nonspecific DNA (red), or Max E-box DNA (blue). DNA sequences are given in Table 2. Samples contained 10 µM protein monomer and 10 µM DNA where appropriate. Each spectrum was averaged twice, and curves were not subjected to smoothing. The buffer control was subtracted from each protein spectrum. Mean residue ellipticities are presented, which account for differences in lengths of proteins.
(TIF)
