# Supplementary figures and images for: Crystal Structure of the Minimalist Max-E47 Protein Chimera
Source: PLoS One. 2012 Feb 28;7(2):e32136. doi: 10.1371/journal.pone.0032136 (PMC3289634; doi:10.1371/journal.pone.0032136)

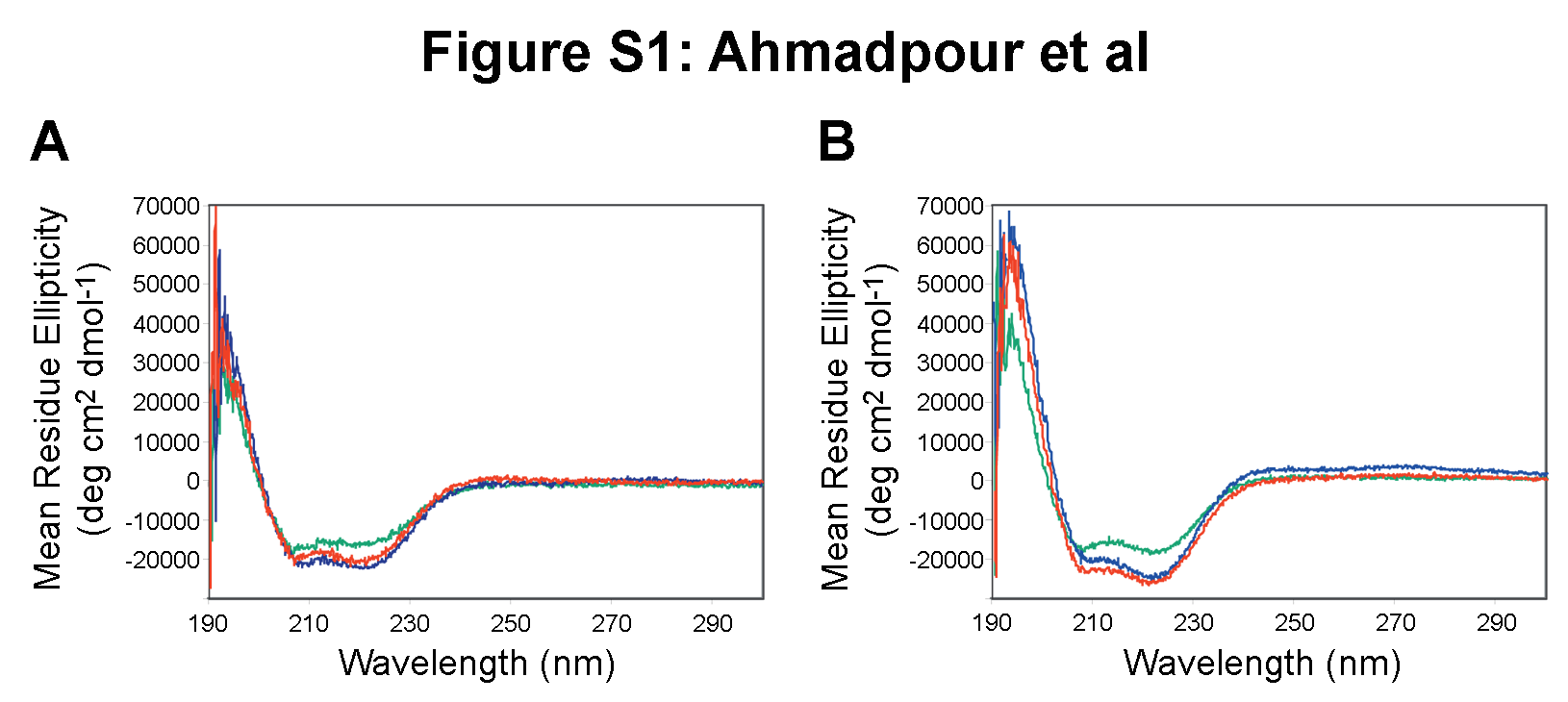

Supplement: Figure S1 — Circular dichroism. Spectra of (A) Max-E47 and (B) MaxbHLHZ in the absence of DNA (green), with nonspecific DNA (red), or Max E-box DNA (blue). DNA sequences are given in Table 2. Samples contained 10 µM protein monomer and 10 µM DNA where appropriate. Each spectrum was averaged twice, and curves were not subjected to smoothing. The buffer control was subtracted from each protein spectrum. Mean residue ellipticities are presented, which account for differences in lengths of proteins. (TIF) [file pone.0032136.s001.tif]
